# Use of WATCH antibiotics prior to presentation to the hospital in rural Burkina Faso

**DOI:** 10.1186/s13756-022-01098-8

**Published:** 2022-04-13

**Authors:** Daniel Valia, Brecht Ingelbeen, Bérenger Kaboré, Ibrahima Karama, Marjan Peeters, Palpouguini Lompo, Erika Vlieghe, Annelies Post, Janneke Cox, Quirijn de Mast, Annie Robert, Marianne A. B. van der Sande, Hector Rodriguez Villalobos, Andre van der Ven, Halidou Tinto, Jan Jacobs

**Affiliations:** 1 Institut de Recherche en Sciences de La Santé, Direction Régional du Centre-Ouest/Clinical Research Unit of Nanoro, Nanoro, Burkina Faso; 2 Institute of Tropical Medicine (ITM), Antwerp, Belgium; 3 Epidemiology and Biostatistics Unit, Institut de Recherche Expérimentale Et Clinique, Université Catholique de Louvain, Brussels, Belgium; 4 Antwerp University Hospital, Antwerp, Belgium; 5 Radboud University Medical Center, Radboud Center for Infectious Diseases, Nijmegen, The Netherlands; 6 Jessa Hospital, Hasselt, Belgium; 7 Julius Center, University Medical Center Utrecht, Utrecht, The Netherlands; 8 Microbiology Unit, Institut de Recherche Expérimentale Et Clinique, Université Catholique de Louvain, Brussels, Belgium; 9 Department of Microbiology, Immunology and Transplantation, KU Leuven, Leuven, Belgium

**Keywords:** Community antibiotic use, Antimicrobial resistance, Burkina Faso, AWaRe

## Abstract

**Background:**

In low- and middle-income countries, the prevalence of antimicrobial resistance (AMR) is increasing. To control AMR, WHO recommends monitoring antibiotic use, in particular Watch antibiotics. These are critically important antibiotics, with restricted use because at risk of becoming ineffective due to increasing AMR. We investigated pre-hospital antibiotic use in rural Burkina Faso.

**Methods:**

During 2016–2017, we collected data from patients aged > 3 months presenting with severe acute fever to the rural hospital of Nanoro Health District, Burkina Faso, including antibiotic use in the two weeks prior to consultation or hospitalization. We analysed reported antibiotic use by applying the WHO Access, Watch, Reserve classification.

**Results:**

Of 920 febrile participants (63.0% ≤ 14 years), pre-hospital antibiotic use was reported by 363 (39.5%). Among these 363, microbiological diagnoses were available for 275 (75.8%) patients, of whom 162 (58.9%) were non-bacterial infections. Use of more than one antibiotic was reported by 58/363 (16.0%) participants. Of 491 self-referred patients who did not previously visit a primary health care center, 131 (26.7%) reported antibiotic use. Of 424 antibiotics reported, 265 (62.5%) were Access and 159 (37.5%) Watch antibiotics. Watch antibiotic use was more frequent among patients > 14 year olds (51.1%) compared to those 0–14 year old (30.7%, *p* < 0.001) and among referrals from the primary health care centers (42.2%) compared to self-referred patients (28.1%, *p* = 0.004). Most frequently reported Watch antibiotics were ceftriaxone (114, 71.7%) and ciprofloxacin (32, 20.1%).

**Conclusion:**

The reported frequent use of Watch group antibiotics among febrile patients prior to presentation to the hospital in rural Burkina Faso highlights the need to develop targeted interventions to improve antibiotic use in community settings as part of strengthening antibiotic stewardship in low- and middle-income countries. This should include facilitating referral, access to qualified prescribers and diagnostic tools in rural primary health care centers.

*Trial registration* ClinicalTrials.gov identifier: NCT02669823. Registration date was February 1, 2016.

## Introduction

The emergence of antimicrobial resistance (AMR) is driven by appropriate and inappropriate use of antimicrobials. Increasing microorganisms’ exposure to antibiotics results in selection pressure, while suboptimal regimen allows selective survival of resistant microorganisms [1, 2]. Globally, mortality attributable to AMR infections has been estimated to increase from 700,000 annual deaths in 2014 to 10 million in 2050 [3]. The World Health Organization's 2015 Global Action Plan on AMR urges a.o. to strengthen surveillance of AMR and antibiotic use, and to optimize the use of antibiotics [4]. A global surveillance network for monitoring concurrently AMR and antibiotic use, the Global Antimicrobial Resistance Surveillance System (GLASS), has since been set up, reporting country-wide AMR prevalence of key human pathogens and antibiotics [5]. To monitor antibiotic use, WHO proposed a classification of antibiotics for human use in three groups, "Access", "Watch" and "Reserve", according to their clinical importance, specific recommendations for their appropriate use, and resistance potential [6]. Antibiotic sales data during 2000–2015 showed that the human use of so-called Watch antibiotics, critically important antibiotics particularly at risk of AMR emergence, was declining in high-income countries, while importantly increasing in middle income countries for which data were available [7, 8].

In sub-Saharan Africa, self-medication with over-the-counter antibiotics from private pharmacies or informal drug stores, without prior prescription by a qualified health worker, is frequent and facilitates uncontrolled antibiotic use [8–11]. To optimize antibiotic use, it is crucial to understand the epidemiology of antibiotic use, in the community as well as in the hospital. Furthermore, also in local primary health care centers, antibiotic prescriptions are not always rational [9, 12]. Peripheral health workers’ poor understanding of the correct use of antibiotics and risks of AMR, limited availability and use of diagnostic tools and difficult access to qualified referral healthcare are likely to be fuelling irrational prescribing of antibiotics [13, 14]. National level antibiotic consumption estimated from wholesale data, as in Burkina Faso, is limited to the official healthcare sector and usually aggregates in- and outpatient use [15]. To our knowledge, no studies so far have investigated community-level antibiotic use. The purpose of this study is to understand community-level antibiotic use in febrile patients prior to presentation to the hospital in a rural district of Burkina Faso.

## Methods

### Study population

From March 23, 2016 to June 30, 2017, the PaluBac study recruited patients aged > 3 months presenting to the Nanoro district hospital with acute fever (tympanic temperature ≥ 38.0 °C) or history of fever in the last 48 h and ≥ 1 symptom(s) of respiratory distress, generalized weakness (prostration), impaired consciousness, seizures (one or more episodes), clinical jaundice, signs of shock, or with suspected severe malaria, invasive bacterial infection, or severe viral infection. A total of 920 patients of whom 344 (37.4%) inpatients and 576 (62.6%) outpatients were included. From March 23 to November 8, 2016, only inpatients were recruited; from November 9, 2016 to June 30, 2017 also outpatients were included. PaluBac evaluated the performance of an automated cell counter to quantify malaria parasitaemia [16]. Our embedded cross-sectional study recorded patients’ pre-hospital antibiotic use.

The study was conducted in the Nanoro Health District in the West-Central region of Burkina Faso, about 90 km from Ouagadougou, the capital city of Burkina Faso. The district has 24 primary health care centers and one district hospital, “Centre Médical avec Antenne Chirurgicale” (CMA). Primary health care centers are meant to be the patients’ first point of contact with the healthcare system, during outpatient consultations. There are no medical doctors attached. More complicated cases are referred from the primary health care centers to the district hospital [17]. The district hospital has hospitalization units, medical doctors and a clinical microbiology laboratory. In the health district, the most frequently reported diagnosis is malaria [18], in children often associated with a community-acquired invasive bacterial co-infection [19, 20]. Malaria is endemic with a *high malaria transmission season* from July to November. This period overlaps with the rainy season, which extends from June to October. The *low malaria transmission season* is from December to June.

### Antibiotics dispensing in Burkina Faso

In Burkina Faso health districts, antibiotics can be obtained from formal public or private drug stores after prescription by a Medical Doctor or a nurse. Importation and quality of antibiotics (and medicines in general) is under the control of the national pharmaceutical regulatory agency [21]. However, some medicines escape from this control and are available over-the-counter in public places such as markets [22]. Also in formal drug stores, antibiotics can be purchased without prior consultation or prescription. In primary health care centers, the only diagnostic tools available to support febrile diseases management are malaria rapid diagnostic tests (RDT). If such tests show a negative result, this often leads to systematic antibiotic prescription [23].

### Data collection

At inclusion, study clinicians completed case report forms with medical history, including the use of antibiotics and antimalarials in the two weeks prior to consultation, which we refer to as *pre-hospital antibiotic use*, the date of symptom onset, and whether the patient was referred or not from a primary health care center. *Referral patients* first attended a primary health care center where a nurse decided about the need for higher-level care and referral to the district hospital, based on clinical symptoms. Proper inpatient care is not possible at a primary health care center, the patient can only be observed for a maximum of 48 h. *Self-referred patients* were those presenting directly at the district hospital without first attending a primary health care center.

### Data analysis

Pre-hospital antibiotics were classified (1) according to WHO's Access, Watch, Reserve (AWaRe) classification and (2) according to products name as stated in the WHO essential medicines list [6]. For the latter, we combined ampicillin and amoxicillin use into one category (“ampicillin or amoxicillin”), and zoomed in on the use of the most prevalently prescribed antibiotic in the Watch group in particular. Anti-tuberculosis drugs were excluded from analysis. We report frequencies of antibiotics used by product name and by AWaRe group, of the use of more than one antibiotic, and compared by age (0–14 years vs. > 14 years), by malaria transmission season, by hospitalisation status after arrival to the hospital (in- vs outpatients) and by referral vs self-referred, using chi-square tests. We stratified the frequencies of pre-hospital antibiotic use by malaria transmission season. Data was analysed with Stata version 14.2 (StataCorp LP, Texas, USA).

## Results

### Characteristics of the study population

Of 1212 screened febrile patients, 920 (75.9%) patients were included in the study and had complete data available about pre-hospital antibiotic use. Of these 920, 344 (37.4%)were hospitalized upon arrival at the hospital and 576 (62.6%) were treated as outpatients. Children aged 0–14 years represented 580 (63.0%) of patients and 533 (57.9%) patients were male. A total of 428 (46.5%) patients were referrals from primary health care centers, the remaining were self-referred (Table 1).

**Table 1:** Characteristics of the study population according to age, gender, pre-hospital antibiotic use, malaria season and care status from March 23, 2016 to June 30, 2017 at the district hospital of Nanoro, Burkina Faso (n = 920)

		Number (n)	Percentage (%)
Age (years)	0–14	580	63.0
	> 14	340	37.0
Gender	Male	533	57.9
	Female	387	42.1
Pre-hospital antibiotic use	Yes	363	39.5
	No	557	60.5
Referrals from primary health care centers*	Yes	428	46.6
	No	491	53.4
Patients in high malaria season	Yes	377	41.0
	No	543	59.0
Hospitalized upon arrival to the hospital	Yes	344	37.4
	No	576	62.6

*Missing data in 1 patient

### Pre-hospital antibiotic use

Pre-hospital antibiotic use was reported by 363/920 (39.5%) patients, of whom 58 (16.0%) used more than one antibiotic. Proportion of use tended to be more prevalent among 0–14 year olds (240, 41.4%) compared to those > 14 (123, 36.2%) (*p* = 0.12). Referral patients significantly more frequently reported pre-hospital antibiotic use (231, 54.0%) than self-referred patients (131, 26.7%, *p* < 0.001). Fewer patients (131, 34.7%) reported antibiotic use during the high malaria transmission season than outside (232, 42.7%, *p* < 0.001).

Microbiological diagnoses (malaria microscopy, blood culture, PCR of nasopharyngeal swabs) were available for 275/363 (75.8%) patients who reported pre-hospital antibiotic use. Of these, 113 (41.1%) were bacterial infections; the other diagnoses were malaria (70, 25.5%), viral infections excluding HIV (85, 30.9%), and HIV (7, 2.6%) HIV.

### AWaRe distribution of antibiotics

Overall, 424 antibiotics were reported by 363 patients: 265 (62.5%) antibiotics belonged to the Access group, 159 (37.5%) to the Watch group and none to the Reserve group. Watch antibiotics were more frequently reported by > 14 year olds (72, 51.1%) than by 0–14 year olds (87, 30.7%, *p* < 0.001) and by referrals (117, 42.2%) compared to self-referred patients (41, 28.1%, *p* = 0.004). There was no difference in the proportion of Watch antibiotics used between the high and low malaria-transmission season (Table 2).

**Table 2:** Bivariate risk factors associated with pre-hospital Watch group antibiotic use among patients reporting pre-hospital antibiotic use from March 23, 2016 to June 30, 2017 at the district hospital of Nanoro, Burkina Faso (424 antibiotics used by 363 patients reporting antibiotic use)

	Number of antibiotics reported (n = 424)	Watch group antibiotic use (n = 159)		Odds ratio (95%CI)
		n	%	
*Age (Years)*				
0–14	283	87	30.7	1
> 14	141	72	51.1	**2.35** (1.55–3.56)
*Malaria season*				
High	146	56	38.4	1
Low	278	103	37.1	0.95 (0.63–1.43)
*Care status*				
Not hospitalized	286	113	39.5	1
Hospitalized	138	46	33.3	0.77 (0.50–1.17)
Referral status*				
Self-referred	146	41	28.1	1
Referred from primary health care centers	277	117	42.2	**1.87** (1.22–2.89)

An association is statistically significant (in bold) when the confidence interval does not include the number 1

*Missing data in 1 patient, n is the number either of all antibiotics reported or Watch group antibiotics reported

### Antibiotics reported

Ampicillin or amoxicillin use (Access) was the most reported antibiotic, accounting for 137/424 (32.3%) of all antibiotics reported. The most frequently reported Watch antibiotics were ceftriaxone (114, 26.9% of all antibiotics reported) and ciprofloxacin (32, 7.5% of all antibiotics reported). Among antibiotics reported by referral patients (n = 277 antibiotics), ceftriaxone was recorded 100 times (36.1%) and ciprofloxacin 12 times (4.3%). Among antibiotics reported by self-referred patients (n = 146), ciprofloxacin was recorded 20 times (13.7%) (Table 3).

**Table 3:** Distribution of pre-hospital antibiotics used for severe febrile illness by WHO AWaRe classification and by referral status from March 23, 2016 to June 30, 2017 at the district hospital of Nanoro, Burkina Faso (424 antibiotics used by 363 patients reporting antibiotic use)

Antibiotics reported	Number of antibiotics	WHO AWaRe classification	Referral status*			
			Referrals		Self-referred	
			n	%	n	%
Phenoxymethylpenicillin	3	Access	3	1.1	0	0.0
Ampicillin or amoxicillin	137	Access	95	34.3	42	28.8
Amoxicillin + clavulanic acid	10	Access	1	0.4	9	6.2
Cefadroxil	2	Access	1	0.4	1	0.7
Trimethoprim + sulfamethoxazole	45	Access	18	6.5	27	18.5
Metronidazole	45	Access	21	7.6	24	16.4
Gentamicin	21	Access	21	7.6	0	0.0
Thiamphenicol	2	Access	0	0.0	2	1.4
Total Access antibiotics	265		160	57.8	105	71.9
Ceftriaxone	114	Watch	100	36.1	13	8.9
Ciprofloxacin	32	Watch	12	4.3	20	13.7
Erythromycin	12	Watch	5	1.8	7	4.8
Cefixime	1	Watch	0	0.0	1	0.7
Total Watch antibiotics	159		117	42.2	41	28.1
Total number of antibiotics	424		277	100	146	100

*For one ceftriaxone course reported, data was missing on referral status. Numbers refer to total numbers of products reported by patients; percentage relates to the total number of antibiotics (n = 277 and n = 146 for referrals and self-referred patients respectively)

## Discussion

Nearly 40% of patients presenting with acute fever to a referral hospital in rural Burkina, reported pre-hospital antibiotic use; more than half of patients who were referred by a primary health care centre, and a quarter of self-referred patients. Nearly 60% of febrile patients with pre-hospital antibiotic use for whom a microbiological diagnosis was available did not have a bacterial infection confirmed.

While empirical first- or second-choice (Access) antibiotics were most frequently used, Watch antibiotics were used by 42% of patients referred from a primary health care center. The high use of Watch antibiotics in referred patients is worrisome particular since ceftriaxone (Watch) is not on the Burkina Faso medicine list for use in primary health care centers and is recommended at this level only in case of a meningitis outbreak (which did not occur during our study period) [24]. Moreover, Watch antibiotics accounted for 28% of antibiotics used by self-referred patients, who presumably self-medicated with antibiotics obtained without prescription at private pharmacies or from informal drug sellers. Use of Watch antibiotics was not associated with severity or seasonality as there was no increased risk for patients admitted compared to those treated as outpatients nor during low-malaria transmission season compared to high-malaria transmission season.

High and increasing use of Watch antibiotics has been observed in other low- and middle-income settings [7, 25, 26]. To optimize antibiotic use, it is important to better understand the origins of and reasons why Watch antibiotics are used, both at primary health care center level and without prescription at the community-level. Our study illustrates the need to monitor antibiotic use among official and informal healthcare providers. Nationwide antibiotic use estimated from official sales data found that, in 2015, 75% of antibiotics used in Burkina Faso were Access and 24% Watch group, achieving the WHO target of at least 60% of antibiotics used to be Access antibiotics [15]. This use of Watch antibiotics is lower than the proportion of Watch antibiotics reported in the present study. In comparison, the frequency of pre-hospital antibiotic use by febrile patients admitted to the Nanoro district hospital in 2012–2013 was lower (28.2%), and the proportion of Access antibiotics used was higher (45.9% amoxicillin or ampicillin vs. 32.3% now and 35.1% trimethoprim + sulfamethoxazole vs. 10.6% now) [19], further confirming the need to halt and reverse this high prevalence of Watch antibiotic use.

Despite ampicillin being the treatment of choice for patients with severe infectious diseases at primary health care center level pending referral, it is increasingly ineffective against *Enterobacterales*. Indeed, most (90.5%) of non-Typhi *Salmonella* and 87.5% of *Escherichia coli* were resistant to ampicillin in Nanoro during 2012–2013 [19]. This could explain (systematic) use of ceftriaxone when—in the absence of a microbiological diagnosis—bloodstream infection is suspected. Rapid referral to the district hospital, where laboratory testing should be available as recommended in the WHO model list of essential in vitro diagnostics [27] is then indicated. Because cephalosporin use has been associated to the emergence of beta-lactamase-producing pathogens, particular caution must be taken to optimize its use [28]. Integrating point-of-care CRP or procalcitonin tests at primary health care center level, to differentiate between bacterial and non-bacterial causes of fever, could be explored [29].

Some caution interpreting these findings is needed. First, the inclusion of outpatients during the second half of the study period might have resulted in some changes in the study population and pre-hospital antibiotic use. However, we observed no difference in the prevalence of antibiotic use, when comparing the two populations from both recruitment periods. Further, data about pre-hospital antibiotic use were missing for a quarter of screened patients. Also, antibiotic use was collected via a survey capturing use during the two weeks before consultation at the hospital, potentially underestimating actual use. Whenever possible, reported antibiotic use was verified from referral forms, patient medical files (healthcare booklet), and antibiotic packaging or blisters. Dosage and duration of antibiotic used were not available and the final diagnoses at the district hospital may not correspond to those that had triggered prescription or self-medication within the two preceding weeks.

## Conclusion

The frequent use of Watch antibiotics in primary health care centers and at community-level in rural Burkina Faso is concerning in a context of increasingly ineffective first-line antibiotics. It points to the need to set up antibiotic use monitoring and antibiotic stewardship in rural communities in low- and middle-income countries (LMIC. As no alternatives for the currently available Watch antibiotics are available nor affordable in many LMIC settings, it is imperative to act to avoid even more alarming levels of resistance in years to come.

## Data Availability

All data generated or analysed during this study are included in this published article and datasets used during the current study are available from the corresponding author on reasonable request.

## Abbreviations

AMR: Antimicrobial resistance
AWaRe: Access, watch, reserve
CMA: Centre Médical avec Antenne Chirurgicale
CRP: C-reactive protein
GLASS: Global antimicrobial resistance surveillance system
HIV: Human immunodeficiency virus
RDT: Rapid diagnostic test
WHO: World Health Organization
